# Crystal structure of bis(5-bromo-1,10-phenanthroline-κ^2^ *N*,*N*′)bis­[di­hydro­bis­(pyrazol-1-yl)borato-κ^2^ *N* ^2^,*N* ^2′^]iron(II) toluene disolvate

**DOI:** 10.1107/S2056989020010361

**Published:** 2020-07-31

**Authors:** Sascha Ossinger, Christian Näther, Felix Tuczek

**Affiliations:** a Institut für Anorganische Chemie, Christian-Albrechts-Universität zu Kiel, Max-Eyth Str. 2, D-24118 Kiel, Germany

**Keywords:** crystal structure, di­hydro­bis­(pyrazol-1-yl)borate phenanthroline, Fe^II^, spin crossover

## Abstract

The structure determination of the title compound was undertaken as part of a project on the modification and synthesis of new spin-crossover (SCO) compounds based on octa­hedral Fe^II^ bis­(pyrazol­yl)borate complexes. In the course of these investigations, the compound [Fe(C_6_H_8_BN_4_)_2_(C_12_H_7_BrN_2_)] was synthesized, for which magnetic measurements revealed an incomplete spin-crossover behaviour. Crystallization of this compound from toluene led to the formation of crystals of the toluene disolvate, [Fe(C_6_H_8_N_4_B)_2_(C_12_H_7_N_2_Br)]·2C_7_H_8_. Its asymmetric unit comprises two discrete metal complex mol­ecules and two toluene solvent mol­ecules. One of the latter is severely disordered and its contribution to the diffracted intensities was removed using the SQUEEZE routine [Spek (2015 ▸). *Acta Cryst*. C**71**, 9–18]. In each complex mol­ecule, the Fe^II^ cation is coordinated by the two N atoms of a 5-bromo-1,10-phenanthroline ligand and by two pairs of N atoms of chelating di­hydro­bis(pyrazol-1-yl)borate ligands in the form of a slightly distorted octa­hedron. The discrete complexes are arranged in columns along the *a-*axis direction with the toluene solvate mol­ecules located between the columns. The 5-bromo-1,10-phenanthroline ligands of neighbouring columns are approximately parallel and are slightly shifted relative to each other, indicating π–π inter­actions.

## Abstract 2

Each of the two unique metal complex mol­ecules in the title compound shows a distorted N_6_ coordination set defined by three pairs of chelating ligands.

## Chemical context   

Spin crossover (SCO) occurs in octa­hedrally coordinated transition-metal complexes with an electron configuration of 3*d* ^4^–3*d* ^7^ and is of extraordinary importance in coordination chemistry and the field of mol­ecular magnetism. Such mat­erials are also of inter­est because of their potential for future applications as mol­ecular switches, in data storage or in spintronics (Gütlich *et al.*, 2013 ▸; Halcrow, 2007 ▸, 2013*b* ▸). SCO compounds can be switched between the paramagnetic high-spin (HS, *S* = 2) and the diamagnetic low-spin state (LS, *S* = 0) by external stimuli such as temperature or light (Gütlich *et al.*, 2013 ▸). Most compounds reported in the literature are based on Fe^II^ in an octa­hedral coordination because, in this case, a very long lifetime of the photochemically excited high-spin state is expected. During the spin-transition, the Fe—*L* (*L* = ligand) bond lengths and also the unit-cell volume change significantly. Therefore, cooperativity effects are of importance, which frequently lead to abrupt spin transitions, very often associated with a hysteresis or a more complicated SCO behaviour (Halcrow, 2007 ▸, 2013*a* ▸). Up to date, hundreds of Fe^II^ SCO complexes have been published (Halcrow, 2007 ▸). Recently, complexes based on organoborate ligands such as [Fe(H_2_B(pz)_2_)_2_(*L*)] (with pz = pyrazole and *L* = di­imine co-ligand) have become of particular inter­est, because they can be evaporated *in vacuo* and therefore allow a facile preparation of thin films (Ruben & Kumar, 2019 ▸; Naggert *et al.*, 2015 ▸; Ossinger *et al.*, 2020*a* ▸).
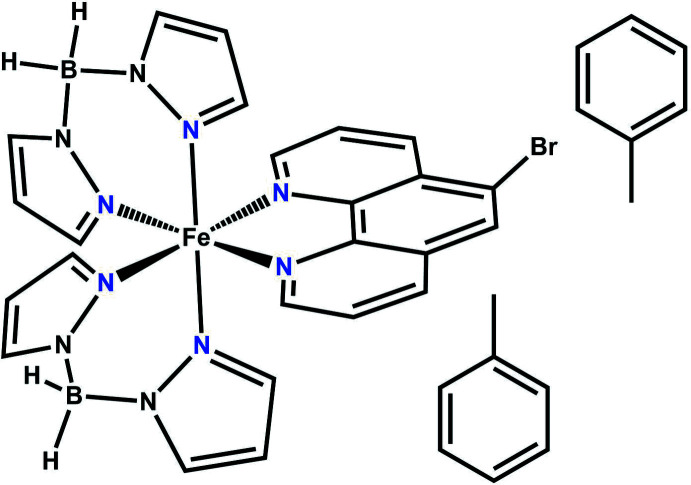


In our own systematic investigations we are inter­ested how a chemical modification of such Fe organoborate complexes influences the SCO behavior in the bulk material and in thin films. This includes functionalization of the neutral di­imine ligand *L* and the pyrazole ligand in iron(II) complexes with general composition [Fe(H_2_B(pz)_2_)_2_(*L*)], which leads to characteristic changes in the spin-transition behaviour in the solid state (Naggert *et al.*, 2015 ▸; Ossinger *et al.*, 2019 ▸, 2020*a* ▸,*b* ▸). Analysis of the crystal structures of these iron(II) bis­(di­hydro­bis­(pyrazol­yl)borate) complexes reveals that most of them are built up of dimers that are linked by inter­molecular π–π inter­actions between the phenyl rings of the co-ligands *L* (Ossinger *et al.*, 2020*b* ▸). We found that the toluene solvate [Fe(H_2_B(pz)_2_)_2_(4,7-dimephen)]·0.5C_7_H_8_ exhibits a short π–π intra-dimer distance (3.507 Å at 293 K and 3.483 Å at 200 K) and consequently the complexes are locked in the high-spin state, whereas for [Fe(H_2_B(pz)_2_)_2_(4,4′-dimebipy)] with a long π–π distance (3.753 Å at 293 K and 3.736 Å at 200 K) complete thermal SCO is observed (Ossinger *et al.*, 2020*b* ▸). Alternatively, for the inter­mediate distances (3.575 Å at 300 K and 3.508 Å at 140 K) found in [Fe(H_2_B(4-CH_3_-pz)_2_)_2_(bipy)] an incomplete spin-crossover is observed (Ossinger *et al.*, 2020*b* ▸). In the course of this project we became inter­ested in the compound [Fe(H_2_B(pz)_2_)_2_(5-Br-phen)] (pz = pyrazole, 5-Br-phen = 5-bromo-1,10-phenanthroline). Magnetic measurements of this new complex revealed an incomplete SCO in the temperature range from 2 to 300 K with only one step during the spin transition (see Fig. S1 in the supporting information). This compound can easily be crystallized from toluene whereby a toluene disolvate, [Fe(H_2_B(pz)_2_)_2_(5-Br-phen)]·2C_7_H_8_, is formed. The crystal structure of this solvate shows dimers that are linked by phenanthroline ligands with an intra-dimer distance of 3.465 (6) Å at 200 K, indicating strong inter­molecular π–π inter­actions (see above). Therefore, the system may be locked in the HS, which would concur with the observed bond lengths at 200 K, reflecting a HS configuration, see *Structural commentary*. Unfortunately, we were not able to prepare large amounts of pure samples of the title compound for magnetic measurements. On the other hand, a comparison of the experimental XRPD pattern of the ansolvate with the simulated pattern of the title complex based on single-crystal data (Fig. S2) reveals that the crystal structure of the disolvate is entirely different from that of the ansolvate. Therefore, we have no information as to whether π–π inter­actions are also present in the ansolvate and, if so, how strong these are. Nevertheless, from the observation of thermal spin-crossover in the latter (albeit in an incomplete fashion), we can conclude that the strength of the π–π inter­actions must be weaker in the ansolvate than in the solvate.

## Structural commentary   

**Figure 1:**
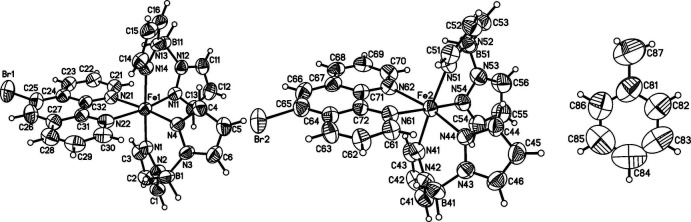
Mol­ecular structures of both crystallographically independent title complexes (left and middle) and of the toluene solvate mol­ecule (right) with atom labelling and displacement ellipsoids drawn at the 50% probability level.

The asymmetric unit of the title compound comprises two discrete complexes of [Fe(H_2_B(pz)_2_)_2_(5-bromo-1,10-phenanthroline)] and two toluene mol­ecules (Fig. 1 ▸). One of the solvent mol­ecules shows severe disorder and was not taken into account in the final model (see *Refinement*). The Fe^II^ cation of each independent complex is distorted octa­hedrally coordinated (Table 1 ▸) by the two N atoms of the chelating 5-bromo-1,10-phenanthroline ligand and by two pairs of N atoms of two chelating di­hydro­bis­(pyrazol-1-yl)borate ligands (Fig. 1 ▸). The two complexes are different regarding their individual bond lengths and angles (Table 1 ▸). The Fe—N bond lengths involving the di­hydro­bis­(pyrazol-1-yl)borate ligand are 2.144 (3)–2.166 (3) or 2.138 (3)–2.205 (3) Å and thus are significantly shorter than those to the 5-bromo-1,10-phenanthroline ligand of 2.204 (3)–2.206 (3) or 2.212 (3)–2.228 (3) Å. The overall bond lengths (average 2.170 Å for complex Fe1, and 2.188 Å for complex Fe2) are in the range expected for Fe^II^ high-spin complexes.

## Supra­molecular features   

**Figure 2:**
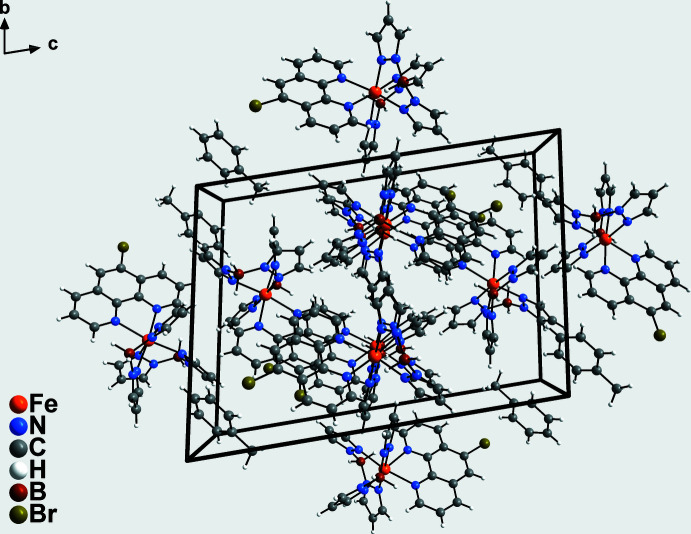
Crystal structure of the title compound in a view along the *a* axis.

**Figure 3:**
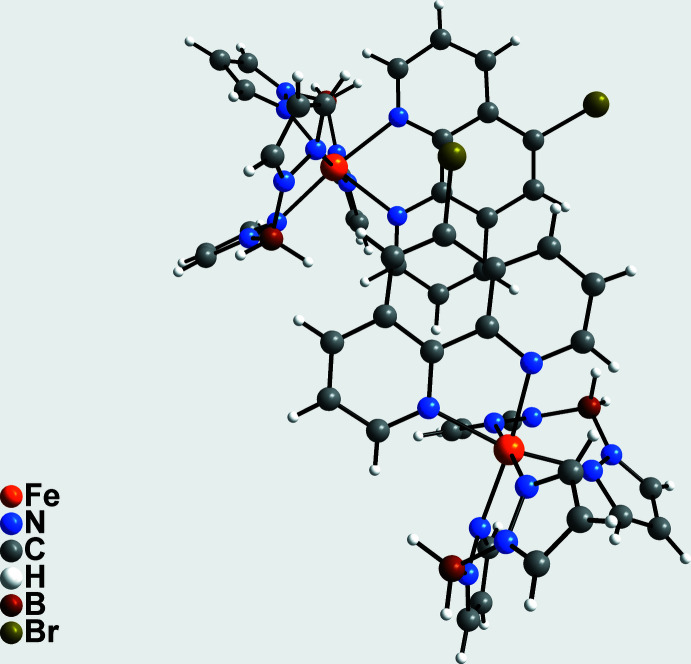
Parts of the crystal structure of the title compound emphasizing the arrangement of the 5-bromo-1,10-phenanthroline ligands.

In the crystal structure, the discrete complexes are arranged into columns that extend along the *a-*axis direction (Fig. 2 ▸). Between these columns, channels are formed in which the toluene solvate mol­ecules are embedded. The planes of the 5-bromo-1,10-phenanthroline ligands of neighbouring columns are approximately parallel with the planes slightly tilted and shifted relative to each other (Fig. 3 ▸). The shortest distance between two parallel-aligned carbon atoms C28 and C64(−*x*, −*y*, −*z* + 1) of neighbouring 5-bromo-1,10-phenanthroline planes is 3.465 (6) Å, indicating strong π–π inter­actions.

## Database survey   

There are at least 21 crystal structures of iron complexes with di­hydro­bis­(pyrazol-1-yl)borate and different co-ligands reported in the literature, which include [Fe(H_2_B(pz)_2_)_2_(phen)] and [Fe(H_2_B(pz)_2_)_2_(2,2′-bipy)] (Real *et al.*, 1997 ▸; Thompson *et al.*, 2004 ▸) as the most well-known complexes. In the others, the co-ligand is replaced by annelated bipyridyl ligands (Kulmaczewski *et al.*, 2014 ▸), various modified diaryl-ethene ligands (Nihei *et al.*, 2013 ▸; Milek *et al.*, 2013 ▸; Mörtel *et al.*, 2017 ▸, 2020 ▸), 4,7-di­methyl­phenanthroline (Naggert *et al.*, 2015 ▸), di­methyl­bipyridine derivatives substituted in the 5,5′ position (Xue *et al.*, 2018 ▸), di­amino­bipyridine (Luo *et al.*, 2016 ▸), chiral (*R*)/(*S*)-4,5-pinenepyridyl-2-pyrazine ligands (Ru *et al.*, 2017 ▸) and further complexes with methyl substituents at the pyrazole unit or co-ligand unit also forming solvate-free or toluene solvate crystals (Ossinger *et al.*, 2019 ▸, 2020*a* ▸,*b* ▸). In all of these complexes, the Fe^II^ cations are coordinated by three bidentate chelate ligands in a more or less distorted octa­hedral environment and show spin-crossover behaviour. Moreover, the structure of the synthetic inter­mediate used for the preparation of the Fe phenanthroline complex, [Fe(H_2_B(pz)_2_)_2_(MeOH)_2_], has also been published (Ossinger *et al.*, 2016 ▸).

## Synthesis and crystallization   

All reactions were carried out in dry solvents and the complexation was carried out under nitro­gen-atmosphere using standard Schlenk techniques or in an M-Braun Labmaster 130 glovebox under argon.

1*H-*Pyrazole, 5-bromo-1,10-phenanthroline and potassium tetra­hydro­borate were purchased from commercial sources and used without further purification. Iron(II) triflate is also commercial available but was purified by the following method: the compound was dissolved in dry methanol (a few ml for a supersaturated solution), filtered off and afterwards the solvent was removed *in vacuo*. Solvents were purchased from commercial sources and purified by distilling over conventional drying agents. K[H_2_B(pz)_2_] was synthesized according to previously reported procedures (Naggert *et al.*, 2015 ▸; Ossinger *et al.*, 2019 ▸, 2020*a* ▸).

**Synthesis of [Fe(H_2_B(pz)_2_)_2_(5-bromo-1,10-phenanthroline]:** To a solution of Fe(OTf)_2_ (353 mg, 1.00 mmol) in methanol (3 ml), a solution of K(H_2_B(pz)_2_) (373 mg, 2.00 mmol) in methanol (5 ml) was added, leading to the formation of a slightly yellow-coloured solution, which was stirred for 15 min at room temperature. A solution of 5-bromo-1,10-phenanthroline (259 mg, 1.00 mmol) in methanol (3 ml) was added dropwise to the reaction mixture. Immediately, the solution turned purple and a purple-coloured precipitate was formed. The solution was stirred for 1 h at room temperature and then the precipitate was filtered off, washed with methanol (7 ml), and dried under reduced pressure. Yield: 373mg [612 µmol, 61% based on Fe(OTf)_2_].

**Elemental analysis** calculated for C_24_H_23_B_2_BrFeN_10_: C 47.34, H 3.81, N 23.00, Br 13.12%, found C 47.11, H 3.92, N 22.97, Br 13.41%.

**HRESI–MS (+) (MeOH):** *m*/*z* (%) = [H_2_B(Hpz)_2_]^+^ calculated 149.09930, found 149.09927 (33), [co-ligand + H]^+^ calculated 258.98654, found 258.98631 (3), [*M* – H_2_B(pz)_2_]^+^ calculated 460.99786, found 460.99758 (25), [*M* + H]^+^ calculated 609.08988, found 609.08946 (6), [*M* – H_2_B(pz)_2_ + co-ligand]^+^ calculated 720.97507, found 720.97455 (100).

**IR (ATR, 298 K):** ν/cm^−1^ = 3105, 3058 (*w*, ν[=C—H]), 2401, 2352 (*m*, ν_asym._[–BH_2_]), 2285 (*m*, ν_sym._[–BH_2_]), 1599 (*w*), 1574 (*w*), 1501 (*m*), 1482 (*w*), 1421 (*m*), 1398 (*m*), 1372 (*w*), 1339 (*w*), 1292 (*m*), 1258 (*w*), 1203 (*m*), 1184 (*m*), 1154 (*s*), 1092 (*m*), 1064 (*m*), 1049 (*s*), 996 (*w*), 978 (*m*), 933 (*m*), 921 (*w*), 880 (*s*), 845 (*w*), 823 (*w*), 804 (*m*), 766 (*s*), 747 (*s*), 731 (*s*), 719 (*m*), 674 (*m*), 637 (*s*), 621 (*m*), 612 (*m*), 421 (*m*).

**Raman (Bulk, 298K):** ν/cm^−1^ = 2781 (*br*, *s*), 1447 (*w*), 1410 (*w*), 1366 (*w*), 1339 (*w*), 1312 (*w*), 1290 (*w*), 1205 (*w*), 1092 (*w*), 1051 (*w*), 758 (*w*), 719 (*w*).

**UV/Vis (KBr, 298K):** λ_max_/nm = 212, 234, 279, 314, 425–702 (*br*).

**Crystallization:** Single crystals of [Fe(H_2_B(pz)_2_)_2_(5-bromo-1,10-phenanthroline]·2C_7_H_8_ were obtained under a nitro­gen atmosphere by dissolving microcrystalline [Fe(H_2_B(pz)_2_)_2_(5-bromo-1,10-phenanthroline] in dry toluene and overlaying with dry *n*-hexane. After a few days, purple-coloured single crystals were obtained that were collected and dried under reduced pressure.

**Experimental details:** Elemental analyses were performed using a vario MICRO cube CHNS element analyser from Elementar. Samples were burned in sealed tin containers by a stream of oxygen. High-resolution ESI mass spectra were recorded on a ThermoFisher Orbitrap spectrometer. IR spectra were recorded on a Bruker Alpha-P ATR–IR Spectrometer. Signal intensities are marked as *s* (strong), *m* (medium), *w* (weak) and *br* (broad). For FT–Raman spectroscopy, a Bruker RAM II-1064 FT–Raman Module, a R510-N/R Nd:YAG-laser (1046 nm, up to 500 mW) and a D418-T/R liquid-nitro­gen-cooled, highly sensitive Ge detector or a Bruker IFS 66 with a FRA 106 unit and a 35 mW NdYAG-LASER (1064 nm) were used. XRPD experiments were performed with a Stoe Transmission Powder Diffraction System (STADI P) with Cu *K*α radiation (λ = 1.5406 Å) that is equipped with position-sensitive detectors (Mythen-K1). UV/vis spectra were recorded with a Cary 5000 spectrometer in transmission geometry. The magnetic measurement was performed at 1 T between 300 and 2 K using a physical property measurement system (PPMS) from Quantum Design. Diamagnetic corrections were applied with the use of Pascal’s constants (Bain & Berry, 2008 ▸).

## Refinement   

Crystal data, data collection and structure refinement details are summarized in Table 2 ▸. C-bound H atoms were positioned with idealized geometry and refined with *U*_iso_(H) = 1.2*U*_eq_(C) using a riding model. B-bound H atoms were located in a difference map, their bond lengths were set to ideal values, and finally they were refined with *U*_iso_(H) = 1.2*U*_eq_(B) using a riding model. The asymmetric unit contains two toluene solvate mol­ecules, of which one is severely disordered. Its contribution to the intensity data was removed using the SQUEEZE (Spek, 2015 ▸) routine in *PLATON* (Spek, 2020 ▸). The disordered toluene mol­ecule was not taken into account in the calculation of the mol­ecular formula and the mol­ecular weight.

## Supplementary Material

Crystal structure: contains datablock(s) I. DOI: 10.1107/S2056989020010361/wm5576sup1.cif

Structure factors: contains datablock(s) I. DOI: 10.1107/S2056989020010361/wm5576Isup2.hkl

Figure S1. ChiT vs T curve from PPMS measurements of [Fe(H2B(pz)2)2(5-Br-phen)] between 2 and 300 K. The curves resulting from cooling and heating the sample almost coincide. DOI: 10.1107/S2056989020010361/wm5576sup3.png

Figure S2. Experimental XRPD pattern of [Fe(H2B(pz)2)2(5-Br-phen)] measuered at rt (a) as well as simulated XRPD pattern calculated from the single crystal structure of [Fe(H2B(pz)2)2(5-Br-phen)]*2 toluene (b) (200 K). DOI: 10.1107/S2056989020010361/wm5576sup4.png

CCDC reference: 2019452

Additional supporting information:  crystallographic information; 3D view; checkCIF report

## Figures and Tables

**Table 1:** Selected bond lengths and angles (Å, °) for the title compound at 200 K

Fe1—N11	2.144 (3)	Fe2—N54	2.138 (3)
Fe1—N4	2.145 (3)	Fe2—N44	2.167 (3)
Fe1—N14	2.154 (3)	Fe2—N51	2.178 (3)
Fe1—N1	2.166 (3)	Fe2—N41	2.205 (3)
Fe1—N21	2.204 (3)	Fe2—N61	2.212 (3)
Fe1—N22	2.206 (3)	Fe2—N62	2.228 (3)
Average bond length	2.170	Average bond length	2.188
			
N11—Fe1—N4	93.27 (13)	N54—Fe2—N44	91.37 (13)
N11—Fe1—N14	88.85 (12)	N54—Fe2—N51	89.35 (12)
N4—Fe1—N14	88.10 (11)	N44—Fe2—N51	89.67 (12)
N11—Fe1—N1	89.44 (12)	N54—Fe2—N41	86.68 (13)
N4—Fe1—N1	90.52 (12)	N44—Fe2—N41	88.27 (12)
N11—Fe1—N21	93.22 (12)	N44—Fe2—N61	93.62 (12)
N14—Fe1—N21	93.76 (11)	N51—Fe2—N61	93.43 (12)
N1—Fe1—N21	87.82 (12)	N41—Fe2—N61	90.72 (12)
N4—Fe1—N22	98.88 (12)	N54—Fe2—N62	100.34 (13)
N14—Fe1—N22	90.32 (12)	N51—Fe2—N62	95.71 (12)
N1—Fe1—N22	91.66 (12)	N41—Fe2—N62	87.13 (12)
N21—Fe1—N22	74.68 (12)	N61—Fe2—N62	74.43 (12)

**Table 2:** Experimental details

Crystal data	
Chemical formula	[Fe(C_6_H_8_BN_4_)_2_(C_12_H_7_BrN_2_)]·C_7_H_8_
*M*_r_	701.04
Crystal system, space group	Triclinic, *P* 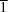
Temperature (K)	200
*a*, *b*, *c* (Å)	10.5035 (4), 15.2782 (5), 20.9003 (7)
α, β, γ (°)	80.266 (3), 86.443 (3), 78.066 (3)
*V* (Å^3^)	3233.0 (2)
*Z*	4
Radiation type	Mo *K*α
μ (mm^−1^)	1.74
Crystal size (mm)	0.14 × 0.10 × 0.08

Data collection	
Diffractometer	Stoe IPDS2
Absorption correction	–
No. of measured, independent and observed [*I* > 2σ(*I*)] reflections	26954, 12583, 8306
*R*_int_	0.037
(sin θ/λ)_max_ (Å^−1^)	0.617

Refinement	
*R*[*F* ^2^ > 2σ(*F* ^2^)], *wR*(*F* ^2^), *S*	0.053, 0.139, 1.02
No. of reflections	12583
No. of parameters	749
H-atom treatment	H-atom parameters constrained
Δρ_max_, Δρ_min_ (e Å^−3^)	0.82, −0.84

Computer programs: *X-AREA* (Stoe & Cie, 2008 ▸), *SHELXT* (Sheldrick, 2015*a* ▸), *SHELXL2014* (Sheldrick, 2015*b* ▸), *DIAMOND* (Brandenburg, 1999 ▸) and *publCIF* (Westrip, 2010 ▸).

## supplementary crystallographic information

### Crystal data

**Table:**

[Fe(C_6_H_8_BN_4_)_2_(C_12_H_7_BrN_2_)]·C_7_H_8_	*Z* = 4
*M**_r_* = 701.04	*F*(000) = 1432
Triclinic, *P*1	*D*_x_ = 1.440 Mg m^−^^3^
*a* = 10.5035 (4) Å	Mo *K*α radiation, λ = 0.71073 Å
*b* = 15.2782 (5) Å	Cell parameters from 26954 reflections
*c* = 20.9003 (7) Å	θ = 1.4–26.0°
α = 80.266 (3)°	µ = 1.74 mm^−^^1^
β = 86.443 (3)°	*T* = 200 K
γ = 78.066 (3)°	Block, purple
*V* = 3233.0 (2) Å^3^	0.14 × 0.10 × 0.08 mm

### Data collection

**Table:**

Stoe IPDS-2 diffractometer	*R*_int_ = 0.037
ω scans	θ_max_ = 26.0°, θ_min_ = 1.4°
26954 measured reflections	*h* = −12→12
12583 independent reflections	*k* = −18→18
8306 reflections with *I* > 2σ(*I*)	*l* = −25→24

### Refinement

**Table:**

Refinement on *F*^2^	0 restraints
Least-squares matrix: full	Hydrogen site location: mixed
*R*[*F*^2^ > 2σ(*F*^2^)] = 0.053	H-atom parameters constrained
*wR*(*F*^2^) = 0.139	*w* = 1/[σ^2^(*F*_o_^2^) + (0.0751*P*)^2^] where *P* = (*F*_o_^2^ + 2*F*_c_^2^)/3
*S* = 1.02	(Δ/σ)_max_ < 0.001
12583 reflections	Δρ_max_ = 0.82 e Å^−^^3^
749 parameters	Δρ_min_ = −0.84 e Å^−^^3^

### Special details

**Table:**

Geometry. All esds (except the esd in the dihedral angle between two l.s. planes) are estimated using the full covariance matrix. The cell esds are taken into account individually in the estimation of esds in distances, angles and torsion angles; correlations between esds in cell parameters are only used when they are defined by crystal symmetry. An approximate (isotropic) treatment of cell esds is used for estimating esds involving l.s. planes.

### Fractional atomic coordinates and isotropic or equivalent isotropic displacement parameters (Å^2^)

**Table:**

	*x*	*y*	*z*	*U*_iso_*/*U*_eq_	
Fe1	0.01316 (5)	−0.25886 (3)	0.51367 (3)	0.03976 (13)	
N1	−0.1584 (3)	−0.1569 (2)	0.52782 (16)	0.0459 (7)	
N2	−0.2794 (3)	−0.1631 (2)	0.51202 (17)	0.0476 (8)	
C1	−0.3628 (4)	−0.0856 (3)	0.5195 (2)	0.0544 (10)	
H1	−0.4535	−0.0731	0.5117	0.065*	
C2	−0.2971 (4)	−0.0280 (3)	0.5399 (2)	0.0586 (11)	
H2	−0.3312	0.0313	0.5491	0.070*	
C3	−0.1695 (4)	−0.0749 (3)	0.5443 (2)	0.0523 (10)	
H3	−0.0994	−0.0519	0.5573	0.063*	
B1	−0.3132 (4)	−0.2520 (3)	0.4989 (3)	0.0545 (12)	
H1A	−0.3094	−0.2980	0.5456	0.065*	
H1B	−0.4139	−0.2356	0.4821	0.065*	
N3	−0.2232 (3)	−0.2899 (2)	0.44479 (17)	0.0487 (8)	
N4	−0.0926 (3)	−0.3169 (2)	0.45283 (16)	0.0459 (7)	
C4	−0.0457 (4)	−0.3549 (3)	0.4006 (2)	0.0505 (9)	
H4	0.0436	−0.3797	0.3930	0.061*	
C5	−0.1434 (4)	−0.3533 (3)	0.3594 (2)	0.0605 (11)	
H5	−0.1361	−0.3759	0.3195	0.073*	
C6	−0.2535 (4)	−0.3116 (3)	0.3892 (2)	0.0605 (11)	
H6	−0.3388	−0.2999	0.3728	0.073*	
N11	0.0743 (3)	−0.1673 (2)	0.43396 (16)	0.0462 (7)	
N12	0.1960 (3)	−0.1776 (2)	0.40582 (16)	0.0479 (7)	
C11	0.1945 (4)	−0.1166 (3)	0.3515 (2)	0.0559 (10)	
H11	0.2674	−0.1094	0.3234	0.067*	
C12	0.0712 (5)	−0.0664 (3)	0.3431 (2)	0.0610 (11)	
H12	0.0416	−0.0184	0.3088	0.073*	
C13	−0.0007 (4)	−0.1003 (3)	0.3949 (2)	0.0545 (10)	
H13	−0.0912	−0.0793	0.4022	0.065*	
B11	0.3162 (4)	−0.2430 (3)	0.4384 (3)	0.0517 (11)	
H11A	0.3992	−0.2431	0.4036	0.062*	
H11B	0.3344	−0.2184	0.4829	0.062*	
N13	0.2871 (3)	−0.3399 (2)	0.45791 (16)	0.0474 (7)	
N14	0.1829 (3)	−0.3589 (2)	0.49551 (16)	0.0462 (7)	
C14	0.1978 (4)	−0.4491 (3)	0.5076 (2)	0.0526 (10)	
H14	0.1390	−0.4813	0.5331	0.063*	
C15	0.3123 (4)	−0.4893 (3)	0.4775 (2)	0.0596 (11)	
H15	0.3457	−0.5521	0.4779	0.071*	
C16	0.3654 (4)	−0.4183 (3)	0.4473 (2)	0.0563 (11)	
H16	0.4450	−0.4233	0.4227	0.068*	
N21	0.1093 (3)	−0.2068 (2)	0.58586 (16)	0.0434 (7)	
N22	−0.0224 (3)	−0.3417 (2)	0.60769 (17)	0.0469 (7)	
C21	0.1738 (4)	−0.1395 (3)	0.5734 (2)	0.0485 (9)	
H21	0.1779	−0.1088	0.5300	0.058*	
C22	0.2357 (4)	−0.1123 (3)	0.6215 (2)	0.0535 (10)	
H22	0.2826	−0.0647	0.6108	0.064*	
C23	0.2285 (4)	−0.1545 (3)	0.6840 (2)	0.0544 (10)	
H23	0.2699	−0.1360	0.7171	0.065*	
C24	0.1596 (4)	−0.2255 (2)	0.6994 (2)	0.0482 (9)	
C25	0.1454 (5)	−0.2758 (3)	0.7631 (2)	0.0615 (11)	
C26	0.0766 (5)	−0.3412 (3)	0.7750 (2)	0.0631 (11)	
H26	0.0683	−0.3718	0.8181	0.076*	
C27	0.0148 (4)	−0.3658 (3)	0.7233 (2)	0.0527 (10)	
C28	−0.0595 (4)	−0.4335 (3)	0.7322 (3)	0.0616 (11)	
H28	−0.0731	−0.4649	0.7745	0.074*	
C29	−0.1123 (4)	−0.4543 (3)	0.6800 (3)	0.0615 (12)	
H29	−0.1627	−0.5002	0.6856	0.074*	
C30	−0.0911 (4)	−0.4071 (3)	0.6183 (2)	0.0556 (10)	
H30	−0.1275	−0.4223	0.5822	0.067*	
C31	0.0291 (3)	−0.3208 (2)	0.65962 (19)	0.0449 (8)	
C32	0.1014 (3)	−0.2495 (2)	0.64774 (19)	0.0417 (8)	
Br1	0.23079 (7)	−0.24781 (4)	0.83229 (3)	0.08442 (19)	
Fe2	0.48657 (5)	0.55322 (4)	0.16744 (3)	0.04260 (14)	
N41	0.3003 (3)	0.6101 (2)	0.21358 (16)	0.0506 (8)	
N42	0.1941 (3)	0.6567 (2)	0.17944 (18)	0.0540 (8)	
C41	0.1160 (5)	0.7097 (3)	0.2169 (3)	0.0664 (12)	
H41	0.0356	0.7486	0.2044	0.080*	
C42	0.1712 (5)	0.6983 (3)	0.2760 (2)	0.0650 (12)	
H42	0.1378	0.7269	0.3122	0.078*	
C43	0.2857 (4)	0.6366 (3)	0.2716 (2)	0.0568 (10)	
H43	0.3463	0.6156	0.3054	0.068*	
B41	0.1731 (5)	0.6448 (4)	0.1090 (3)	0.0592 (12)	
H41A	0.0756	0.6859	0.0980	0.071*	
H41B	0.1759	0.5742	0.1031	0.071*	
N43	0.2882 (3)	0.6726 (2)	0.06536 (17)	0.0528 (8)	
N44	0.4142 (3)	0.6303 (2)	0.07557 (15)	0.0469 (7)	
C44	0.4863 (4)	0.6724 (3)	0.0307 (2)	0.0545 (10)	
H44	0.5785	0.6573	0.0271	0.065*	
C45	0.4096 (5)	0.7404 (4)	−0.0094 (2)	0.0706 (13)	
H45	0.4367	0.7798	−0.0455	0.085*	
C46	0.2849 (5)	0.7390 (3)	0.0141 (2)	0.0683 (13)	
H46	0.2084	0.7787	−0.0031	0.082*	
N51	0.6733 (3)	0.5079 (2)	0.11990 (16)	0.0472 (7)	
N52	0.7812 (3)	0.5414 (2)	0.12632 (17)	0.0511 (8)	
C51	0.6983 (4)	0.4628 (3)	0.0693 (2)	0.0537 (10)	
H51	0.6396	0.4321	0.0537	0.064*	
C52	0.8216 (4)	0.4674 (3)	0.0430 (2)	0.0620 (11)	
H52	0.8633	0.4413	0.0070	0.074*	
C53	0.8700 (4)	0.5178 (3)	0.0802 (2)	0.0585 (11)	
H53	0.9533	0.5336	0.0743	0.070*	
B51	0.7952 (5)	0.5886 (4)	0.1847 (3)	0.0562 (12)	
H51A	0.7904	0.5422	0.2308	0.067*	
H51B	0.8896	0.6093	0.1749	0.067*	
N53	0.6854 (3)	0.6724 (2)	0.18431 (19)	0.0569 (9)	
N54	0.5571 (3)	0.6657 (2)	0.19003 (18)	0.0522 (8)	
C54	0.4884 (5)	0.7498 (3)	0.1891 (3)	0.0634 (12)	
H54	0.3961	0.7655	0.1913	0.076*	
C55	0.5697 (5)	0.8105 (3)	0.1845 (3)	0.0853 (17)	
H55	0.5459	0.8741	0.1839	0.102*	
C56	0.6935 (5)	0.7587 (3)	0.1808 (3)	0.0807 (16)	
H56	0.7720	0.7812	0.1764	0.097*	
N61	0.4111 (3)	0.4326 (2)	0.15460 (15)	0.0458 (7)	
N62	0.5271 (3)	0.4562 (2)	0.25960 (15)	0.0472 (7)	
C61	0.3550 (4)	0.4223 (3)	0.1025 (2)	0.0521 (9)	
H61	0.3615	0.4638	0.0636	0.063*	
C62	0.2870 (4)	0.3537 (3)	0.1015 (2)	0.0574 (10)	
H62	0.2464	0.3495	0.0631	0.069*	
C63	0.2793 (4)	0.2923 (3)	0.1566 (2)	0.0562 (10)	
H63	0.2335	0.2448	0.1568	0.067*	
C64	0.3394 (4)	0.2999 (3)	0.2131 (2)	0.0488 (9)	
C65	0.3370 (4)	0.2405 (3)	0.2740 (2)	0.0559 (10)	
C66	0.4008 (4)	0.2491 (3)	0.3257 (2)	0.0576 (11)	
H66	0.3998	0.2068	0.3646	0.069*	
C67	0.4698 (4)	0.3205 (3)	0.32275 (19)	0.0506 (9)	
C68	0.5378 (4)	0.3337 (3)	0.3750 (2)	0.0542 (10)	
H68	0.5418	0.2925	0.4147	0.065*	
C69	0.5977 (4)	0.4052 (3)	0.3686 (2)	0.0569 (11)	
H69	0.6453	0.4138	0.4035	0.068*	
C70	0.5891 (4)	0.4663 (3)	0.3104 (2)	0.0559 (10)	
H70	0.6294	0.5172	0.3071	0.067*	
C71	0.4697 (4)	0.3835 (3)	0.26542 (19)	0.0453 (8)	
C72	0.4050 (3)	0.3714 (2)	0.20966 (18)	0.0424 (8)	
Br2	0.23735 (6)	0.14820 (3)	0.28221 (3)	0.07646 (17)	
C81	0.2205 (8)	0.0581 (5)	0.0990 (5)	0.112 (2)	
C82	0.1121 (7)	0.1262 (5)	0.0968 (4)	0.110 (2)	
H82	0.0460	0.1233	0.1298	0.132*	
C83	0.0989 (10)	0.1987 (6)	0.0468 (6)	0.122 (3)	
H83	0.0237	0.2457	0.0459	0.147*	
C84	0.1909 (13)	0.2042 (7)	−0.0010 (5)	0.136 (3)	
H84	0.1794	0.2533	−0.0360	0.163*	
C85	0.3007 (11)	0.1379 (9)	0.0019 (5)	0.142 (3)	
H85	0.3676	0.1423	−0.0305	0.170*	
C86	0.3155 (9)	0.0636 (7)	0.0519 (5)	0.131 (3)	
H86	0.3914	0.0172	0.0531	0.157*	
C87	0.2347 (11)	−0.0239 (7)	0.1542 (5)	0.164 (4)	
H87A	0.2381	−0.0793	0.1358	0.245*	
H87B	0.1600	−0.0156	0.1846	0.245*	
H87C	0.3151	−0.0289	0.1772	0.245*	

### Atomic displacement parameters (Å^2^)

**Table:**

	*U*^11^	*U*^22^	*U*^33^	*U*^12^	*U*^13^	*U*^23^
Fe1	0.0376 (3)	0.0402 (3)	0.0424 (3)	−0.0073 (2)	−0.0043 (2)	−0.0084 (2)
N1	0.0462 (17)	0.0437 (17)	0.0485 (19)	−0.0089 (13)	−0.0012 (14)	−0.0091 (14)
N2	0.0385 (16)	0.0447 (17)	0.057 (2)	0.0007 (13)	−0.0043 (14)	−0.0103 (15)
C1	0.047 (2)	0.050 (2)	0.059 (3)	0.0018 (18)	0.0014 (18)	−0.0013 (19)
C2	0.062 (2)	0.041 (2)	0.069 (3)	−0.0008 (19)	0.005 (2)	−0.013 (2)
C3	0.061 (2)	0.040 (2)	0.057 (3)	−0.0091 (17)	0.0024 (19)	−0.0115 (18)
B1	0.036 (2)	0.063 (3)	0.067 (3)	−0.008 (2)	−0.002 (2)	−0.021 (2)
N3	0.0371 (15)	0.0561 (19)	0.056 (2)	−0.0081 (14)	−0.0088 (14)	−0.0170 (16)
N4	0.0414 (16)	0.0502 (18)	0.0467 (19)	−0.0069 (13)	−0.0038 (13)	−0.0114 (14)
C4	0.053 (2)	0.055 (2)	0.046 (2)	−0.0106 (18)	0.0007 (18)	−0.0129 (18)
C5	0.068 (3)	0.069 (3)	0.048 (2)	−0.015 (2)	−0.005 (2)	−0.016 (2)
C6	0.055 (2)	0.068 (3)	0.062 (3)	−0.013 (2)	−0.017 (2)	−0.015 (2)
N11	0.0419 (16)	0.0465 (17)	0.0507 (19)	−0.0087 (13)	−0.0038 (14)	−0.0078 (14)
N12	0.0497 (18)	0.0521 (18)	0.0451 (19)	−0.0165 (14)	−0.0056 (14)	−0.0075 (15)
C11	0.068 (3)	0.055 (2)	0.048 (2)	−0.022 (2)	−0.002 (2)	−0.0059 (19)
C12	0.076 (3)	0.051 (2)	0.054 (3)	−0.014 (2)	−0.015 (2)	0.0042 (19)
C13	0.054 (2)	0.052 (2)	0.055 (3)	−0.0037 (18)	−0.0157 (19)	−0.0066 (19)
B11	0.038 (2)	0.061 (3)	0.057 (3)	−0.013 (2)	−0.0049 (19)	−0.008 (2)
N13	0.0381 (16)	0.0559 (19)	0.0487 (19)	−0.0059 (14)	−0.0055 (14)	−0.0125 (15)
N14	0.0426 (16)	0.0486 (18)	0.0495 (19)	−0.0113 (14)	−0.0058 (14)	−0.0093 (14)
C14	0.052 (2)	0.046 (2)	0.061 (3)	−0.0097 (17)	−0.0144 (19)	−0.0076 (18)
C15	0.055 (2)	0.054 (2)	0.070 (3)	0.000 (2)	−0.015 (2)	−0.019 (2)
C16	0.048 (2)	0.061 (3)	0.057 (3)	0.007 (2)	−0.0098 (19)	−0.020 (2)
N21	0.0451 (16)	0.0373 (15)	0.0485 (19)	−0.0108 (13)	−0.0042 (13)	−0.0045 (13)
N22	0.0420 (16)	0.0418 (16)	0.059 (2)	−0.0119 (13)	0.0014 (14)	−0.0110 (15)
C21	0.053 (2)	0.0401 (19)	0.054 (2)	−0.0121 (16)	−0.0038 (18)	−0.0055 (17)
C22	0.055 (2)	0.042 (2)	0.068 (3)	−0.0152 (17)	−0.007 (2)	−0.0118 (19)
C23	0.057 (2)	0.047 (2)	0.062 (3)	−0.0083 (18)	−0.012 (2)	−0.016 (2)
C24	0.048 (2)	0.0402 (19)	0.056 (2)	−0.0033 (16)	−0.0055 (17)	−0.0116 (17)
C25	0.078 (3)	0.053 (2)	0.051 (3)	−0.005 (2)	−0.010 (2)	−0.008 (2)
C26	0.088 (3)	0.051 (2)	0.048 (3)	−0.015 (2)	−0.002 (2)	−0.0018 (19)
C27	0.057 (2)	0.042 (2)	0.056 (3)	−0.0081 (17)	0.0019 (19)	−0.0032 (18)
C28	0.067 (3)	0.046 (2)	0.068 (3)	−0.012 (2)	0.009 (2)	−0.002 (2)
C29	0.057 (2)	0.041 (2)	0.085 (3)	−0.0126 (18)	0.011 (2)	−0.007 (2)
C30	0.049 (2)	0.046 (2)	0.074 (3)	−0.0113 (17)	0.004 (2)	−0.016 (2)
C31	0.0441 (19)	0.0390 (19)	0.050 (2)	−0.0039 (15)	−0.0042 (16)	−0.0068 (16)
C32	0.0413 (18)	0.0370 (18)	0.046 (2)	−0.0054 (14)	−0.0040 (15)	−0.0071 (16)
Br1	0.1226 (5)	0.0752 (3)	0.0588 (3)	−0.0206 (3)	−0.0276 (3)	−0.0097 (2)
Fe2	0.0480 (3)	0.0463 (3)	0.0376 (3)	−0.0182 (2)	−0.0029 (2)	−0.0068 (2)
N41	0.0559 (19)	0.062 (2)	0.0398 (18)	−0.0210 (16)	0.0001 (14)	−0.0130 (15)
N42	0.0470 (18)	0.062 (2)	0.058 (2)	−0.0156 (16)	0.0012 (16)	−0.0188 (17)
C41	0.060 (3)	0.067 (3)	0.078 (3)	−0.018 (2)	0.014 (2)	−0.029 (3)
C42	0.075 (3)	0.070 (3)	0.062 (3)	−0.030 (2)	0.017 (2)	−0.031 (2)
C43	0.069 (3)	0.063 (3)	0.046 (2)	−0.026 (2)	0.006 (2)	−0.016 (2)
B41	0.048 (2)	0.080 (3)	0.056 (3)	−0.021 (2)	−0.003 (2)	−0.017 (3)
N43	0.0523 (19)	0.060 (2)	0.0454 (19)	−0.0111 (16)	−0.0082 (15)	−0.0053 (16)
N44	0.0467 (17)	0.0537 (18)	0.0411 (18)	−0.0134 (14)	−0.0053 (14)	−0.0044 (14)
C44	0.062 (2)	0.057 (2)	0.044 (2)	−0.020 (2)	−0.0031 (19)	0.0031 (19)
C45	0.080 (3)	0.074 (3)	0.054 (3)	−0.022 (3)	−0.006 (2)	0.007 (2)
C46	0.070 (3)	0.070 (3)	0.058 (3)	−0.004 (2)	−0.012 (2)	0.002 (2)
N51	0.0515 (18)	0.0530 (18)	0.0429 (18)	−0.0198 (15)	−0.0022 (14)	−0.0117 (15)
N52	0.0460 (17)	0.061 (2)	0.049 (2)	−0.0177 (15)	−0.0038 (15)	−0.0085 (16)
C51	0.059 (2)	0.056 (2)	0.048 (2)	−0.0129 (19)	−0.0038 (19)	−0.0123 (19)
C52	0.063 (3)	0.077 (3)	0.047 (2)	−0.014 (2)	0.006 (2)	−0.013 (2)
C53	0.049 (2)	0.079 (3)	0.046 (2)	−0.018 (2)	0.0035 (18)	−0.004 (2)
B51	0.048 (2)	0.073 (3)	0.053 (3)	−0.014 (2)	−0.007 (2)	−0.018 (2)
N53	0.0538 (19)	0.061 (2)	0.066 (2)	−0.0250 (16)	−0.0064 (17)	−0.0174 (18)
N54	0.0512 (18)	0.0498 (19)	0.062 (2)	−0.0197 (15)	−0.0047 (16)	−0.0130 (16)
C54	0.067 (3)	0.046 (2)	0.082 (3)	−0.017 (2)	−0.004 (2)	−0.017 (2)
C55	0.083 (3)	0.051 (3)	0.130 (5)	−0.025 (3)	−0.007 (3)	−0.022 (3)
C56	0.070 (3)	0.063 (3)	0.123 (5)	−0.031 (2)	−0.009 (3)	−0.028 (3)
N61	0.0527 (18)	0.0517 (18)	0.0365 (17)	−0.0157 (14)	−0.0018 (13)	−0.0097 (14)
N62	0.0474 (17)	0.0571 (19)	0.0395 (18)	−0.0122 (15)	−0.0037 (14)	−0.0117 (15)
C61	0.063 (2)	0.060 (2)	0.039 (2)	−0.0227 (19)	−0.0032 (18)	−0.0100 (18)
C62	0.070 (3)	0.069 (3)	0.043 (2)	−0.031 (2)	−0.0003 (19)	−0.014 (2)
C63	0.068 (3)	0.058 (2)	0.052 (3)	−0.032 (2)	0.003 (2)	−0.014 (2)
C64	0.051 (2)	0.048 (2)	0.048 (2)	−0.0107 (17)	0.0050 (17)	−0.0111 (18)
C65	0.063 (2)	0.049 (2)	0.054 (3)	−0.0111 (19)	0.011 (2)	−0.0066 (19)
C66	0.073 (3)	0.046 (2)	0.047 (2)	−0.004 (2)	0.004 (2)	−0.0006 (18)
C67	0.052 (2)	0.051 (2)	0.042 (2)	0.0021 (18)	0.0013 (17)	−0.0050 (17)
C68	0.051 (2)	0.062 (3)	0.042 (2)	0.001 (2)	−0.0052 (17)	−0.0010 (19)
C69	0.049 (2)	0.079 (3)	0.039 (2)	−0.002 (2)	−0.0041 (17)	−0.012 (2)
C70	0.055 (2)	0.072 (3)	0.045 (2)	−0.018 (2)	−0.0037 (18)	−0.016 (2)
C71	0.0457 (19)	0.049 (2)	0.040 (2)	−0.0071 (16)	−0.0005 (16)	−0.0087 (17)
C72	0.0451 (19)	0.0423 (19)	0.040 (2)	−0.0085 (15)	0.0040 (15)	−0.0085 (16)
Br2	0.0981 (4)	0.0581 (3)	0.0763 (4)	−0.0337 (3)	0.0113 (3)	−0.0022 (2)
C81	0.101 (5)	0.096 (5)	0.137 (7)	−0.012 (4)	−0.028 (5)	−0.015 (5)
C82	0.098 (5)	0.100 (5)	0.139 (7)	−0.027 (4)	−0.028 (4)	−0.026 (5)
C83	0.128 (7)	0.091 (5)	0.156 (9)	−0.020 (5)	−0.069 (6)	−0.023 (6)
C84	0.184 (10)	0.118 (7)	0.120 (8)	−0.063 (7)	−0.055 (7)	−0.004 (6)
C85	0.146 (8)	0.165 (9)	0.126 (8)	−0.055 (7)	0.003 (6)	−0.027 (7)
C86	0.106 (6)	0.135 (7)	0.143 (8)	−0.008 (5)	−0.001 (6)	−0.022 (6)
C87	0.168 (9)	0.136 (8)	0.170 (10)	−0.012 (7)	−0.034 (8)	0.009 (7)

### Geometric parameters (Å, º)

**Table:**

Fe1—N11	2.144 (3)	Fe2—N61	2.212 (3)
Fe1—N4	2.145 (3)	Fe2—N62	2.228 (3)
Fe1—N14	2.154 (3)	N41—C43	1.332 (5)
Fe1—N1	2.166 (3)	N41—N42	1.363 (5)
Fe1—N21	2.204 (3)	N42—C41	1.342 (5)
Fe1—N22	2.206 (3)	N42—B41	1.548 (6)
N1—C3	1.335 (5)	C41—C42	1.365 (7)
N1—N2	1.359 (4)	C42—C43	1.375 (6)
N2—C1	1.345 (5)	B41—N43	1.556 (6)
N2—B1	1.544 (6)	N43—C46	1.342 (6)
C1—C2	1.360 (6)	N43—N44	1.360 (4)
C2—C3	1.384 (6)	N44—C44	1.332 (5)
B1—N3	1.540 (6)	C44—C45	1.370 (6)
N3—C6	1.334 (5)	C45—C46	1.374 (7)
N3—N4	1.361 (4)	N51—C51	1.341 (5)
N4—C4	1.342 (5)	N51—N52	1.358 (5)
C4—C5	1.373 (6)	N52—C53	1.340 (5)
C5—C6	1.368 (6)	N52—B51	1.548 (6)
N11—C13	1.340 (5)	C51—C52	1.386 (6)
N11—N12	1.364 (4)	C52—C53	1.368 (7)
N12—C11	1.340 (5)	B51—N53	1.536 (6)
N12—B11	1.552 (5)	N53—C56	1.328 (6)
C11—C12	1.367 (6)	N53—N54	1.368 (5)
C12—C13	1.373 (7)	N54—C54	1.335 (5)
B11—N13	1.555 (6)	C54—C55	1.372 (7)
N13—C16	1.350 (5)	C55—C56	1.381 (7)
N13—N14	1.359 (5)	N61—C61	1.317 (5)
N14—C14	1.337 (5)	N61—C72	1.362 (5)
C14—C15	1.397 (6)	N62—C70	1.326 (5)
C15—C16	1.364 (7)	N62—C71	1.354 (5)
N21—C21	1.326 (5)	C61—C62	1.387 (6)
N21—C32	1.352 (5)	C62—C63	1.367 (6)
N22—C30	1.331 (5)	C63—C64	1.404 (6)
N22—C31	1.355 (5)	C64—C72	1.397 (6)
C21—C22	1.394 (6)	C64—C65	1.437 (6)
C22—C23	1.359 (6)	C65—C66	1.343 (6)
C23—C24	1.408 (6)	C65—Br2	1.902 (5)
C24—C32	1.408 (5)	C66—C67	1.421 (6)
C24—C25	1.436 (6)	C67—C71	1.404 (6)
C25—C26	1.333 (7)	C67—C68	1.405 (6)
C25—Br1	1.899 (5)	C68—C69	1.353 (7)
C26—C27	1.433 (7)	C69—C70	1.398 (6)
C27—C28	1.401 (6)	C71—C72	1.441 (5)
C27—C31	1.406 (6)	C81—C86	1.362 (12)
C28—C29	1.366 (7)	C81—C82	1.371 (10)
C29—C30	1.395 (7)	C81—C87	1.542 (12)
C31—C32	1.432 (5)	C82—C83	1.380 (12)
Fe2—N54	2.138 (3)	C83—C84	1.350 (13)
Fe2—N44	2.167 (3)	C84—C85	1.365 (13)
Fe2—N51	2.178 (3)	C85—C86	1.396 (14)
Fe2—N41	2.205 (3)		
			
N11—Fe1—N4	93.27 (13)	N44—Fe2—N41	88.27 (12)
N11—Fe1—N14	88.85 (12)	N51—Fe2—N41	175.48 (13)
N4—Fe1—N14	88.10 (11)	N54—Fe2—N61	174.30 (13)
N11—Fe1—N1	89.44 (12)	N44—Fe2—N61	93.62 (12)
N4—Fe1—N1	90.52 (12)	N51—Fe2—N61	93.43 (12)
N14—Fe1—N1	177.74 (13)	N41—Fe2—N61	90.72 (12)
N11—Fe1—N21	93.22 (12)	N54—Fe2—N62	100.34 (13)
N4—Fe1—N21	173.29 (12)	N44—Fe2—N62	167.13 (12)
N14—Fe1—N21	93.76 (11)	N51—Fe2—N62	95.71 (12)
N1—Fe1—N21	87.82 (12)	N41—Fe2—N62	87.13 (12)
N11—Fe1—N22	167.79 (12)	N61—Fe2—N62	74.43 (12)
N4—Fe1—N22	98.88 (12)	C43—N41—N42	106.1 (3)
N14—Fe1—N22	90.32 (12)	C43—N41—Fe2	125.8 (3)
N1—Fe1—N22	91.66 (12)	N42—N41—Fe2	123.4 (2)
N21—Fe1—N22	74.68 (12)	C41—N42—N41	109.3 (4)
C3—N1—N2	106.4 (3)	C41—N42—B41	128.3 (4)
C3—N1—Fe1	130.4 (3)	N41—N42—B41	122.5 (3)
N2—N1—Fe1	122.8 (2)	N42—C41—C42	108.7 (4)
C1—N2—N1	108.9 (3)	C41—C42—C43	105.0 (4)
C1—N2—B1	127.4 (3)	N41—C43—C42	110.9 (4)
N1—N2—B1	123.0 (3)	N42—B41—N43	108.3 (3)
N2—C1—C2	109.3 (4)	C46—N43—N44	108.7 (4)
C1—C2—C3	104.7 (3)	C46—N43—B41	128.7 (4)
N1—C3—C2	110.7 (4)	N44—N43—B41	122.7 (3)
N3—B1—N2	110.5 (4)	C44—N44—N43	106.7 (3)
C6—N3—N4	109.1 (3)	C44—N44—Fe2	124.2 (3)
C6—N3—B1	129.6 (3)	N43—N44—Fe2	124.5 (2)
N4—N3—B1	120.9 (3)	N44—C44—C45	111.0 (4)
C4—N4—N3	105.8 (3)	C44—C45—C46	104.6 (4)
C4—N4—Fe1	126.8 (3)	N43—C46—C45	109.1 (4)
N3—N4—Fe1	122.9 (2)	C51—N51—N52	106.3 (3)
N4—C4—C5	111.3 (4)	C51—N51—Fe2	128.5 (3)
C6—C5—C4	104.1 (4)	N52—N51—Fe2	123.3 (2)
N3—C6—C5	109.8 (4)	C53—N52—N51	109.4 (3)
C13—N11—N12	106.2 (3)	C53—N52—B51	128.9 (4)
C13—N11—Fe1	127.8 (3)	N51—N52—B51	121.3 (3)
N12—N11—Fe1	125.0 (2)	N51—C51—C52	110.5 (4)
C11—N12—N11	109.0 (3)	C53—C52—C51	104.6 (4)
C11—N12—B11	127.6 (4)	N52—C53—C52	109.2 (4)
N11—N12—B11	123.1 (3)	N53—B51—N52	109.3 (3)
N12—C11—C12	109.2 (4)	C56—N53—N54	109.1 (4)
C11—C12—C13	104.9 (4)	C56—N53—B51	129.1 (4)
N11—C13—C12	110.7 (4)	N54—N53—B51	121.8 (3)
N12—B11—N13	110.0 (3)	C54—N54—N53	106.5 (3)
C16—N13—N14	109.0 (3)	C54—N54—Fe2	126.1 (3)
C16—N13—B11	126.7 (3)	N53—N54—Fe2	123.6 (2)
N14—N13—B11	123.9 (3)	N54—C54—C55	110.6 (4)
C14—N14—N13	106.8 (3)	C54—C55—C56	104.8 (4)
C14—N14—Fe1	128.2 (3)	N53—C56—C55	109.0 (4)
N13—N14—Fe1	124.0 (2)	C61—N61—C72	118.3 (3)
N14—C14—C15	110.3 (4)	C61—N61—Fe2	125.9 (3)
C16—C15—C14	104.6 (4)	C72—N61—Fe2	114.9 (2)
N13—C16—C15	109.3 (4)	C70—N62—C71	117.7 (4)
C21—N21—C32	118.8 (3)	C70—N62—Fe2	127.5 (3)
C21—N21—Fe1	125.7 (3)	C71—N62—Fe2	114.5 (2)
C32—N21—Fe1	115.4 (2)	N61—C61—C62	123.2 (4)
C30—N22—C31	117.8 (4)	C63—C62—C61	119.1 (4)
C30—N22—Fe1	126.9 (3)	C62—C63—C64	119.6 (4)
C31—N22—Fe1	115.2 (3)	C72—C64—C63	117.4 (4)
N21—C21—C22	122.4 (4)	C72—C64—C65	117.8 (4)
C23—C22—C21	119.6 (4)	C63—C64—C65	124.9 (4)
C22—C23—C24	119.8 (4)	C66—C65—C64	122.1 (4)
C32—C24—C23	116.9 (4)	C66—C65—Br2	119.1 (3)
C32—C24—C25	117.7 (4)	C64—C65—Br2	118.8 (3)
C23—C24—C25	125.4 (4)	C65—C66—C67	120.8 (4)
C26—C25—C24	122.7 (4)	C71—C67—C68	116.8 (4)
C26—C25—Br1	119.4 (4)	C71—C67—C66	119.5 (4)
C24—C25—Br1	117.9 (4)	C68—C67—C66	123.7 (4)
C25—C26—C27	120.6 (4)	C69—C68—C67	119.8 (4)
C28—C27—C31	117.1 (4)	C68—C69—C70	119.6 (4)
C28—C27—C26	123.7 (4)	N62—C70—C69	122.7 (4)
C31—C27—C26	119.1 (4)	N62—C71—C67	123.3 (4)
C29—C28—C27	120.0 (4)	N62—C71—C72	117.7 (3)
C28—C29—C30	118.9 (4)	C67—C71—C72	119.0 (4)
N22—C30—C29	123.2 (4)	N61—C72—C64	122.5 (4)
N22—C31—C27	123.0 (4)	N61—C72—C71	116.9 (3)
N22—C31—C32	117.3 (3)	C64—C72—C71	120.7 (4)
C27—C31—C32	119.7 (4)	C86—C81—C82	119.3 (8)
N21—C32—C24	122.5 (4)	C86—C81—C87	120.3 (8)
N21—C32—C31	117.3 (3)	C82—C81—C87	120.4 (9)
C24—C32—C31	120.2 (4)	C81—C82—C83	120.1 (9)
N54—Fe2—N44	91.37 (13)	C84—C83—C82	121.2 (9)
N54—Fe2—N51	89.35 (12)	C83—C84—C85	119.0 (10)
N44—Fe2—N51	89.67 (12)	C84—C85—C86	120.7 (10)
N54—Fe2—N41	86.68 (13)	C81—C86—C85	119.7 (9)

## References

[bb1] Bain, G. A. & Berry, J. F. (2008). *J. Chem. Educ.* **85**, 532–536.

[bb2] Brandenburg, K. (1999). *DIAMOND*. Crystal Impact GbR, Bonn, Germany.

[bb3] Gütlich, P., Gaspar, A. B. & Garcia, Y. (2013). *Beilstein J. Org. Chem.* **9**, 342–391. 10.3762/bjoc.9.39 PMC3596041 23504535

[bb4] Halcrow, M. A. (2007). *Polyhedron*, **26**, 3523–3576.

[bb5] Halcrow, M. A. (2013*a*). *Chem. Commun.* **49**, 10890–10892. 10.1039/c3cc44171g 24145383

[bb6] Halcrow, M. A. (2013*b*). *Spin-Crossover Materials*. Oxford, UK: John Wiley and Sons Ltd.

[bb7] Kulmaczewski, R., Shepherd, H. J., Cespedes, O. & Halcrow, M. A. (2014). *Inorg. Chem.* **53**, 9809–9817. 10.1021/ic501402q 25171341

[bb8] Luo, Y.-H., Liu, Q.-L., Yang, L.-J., Sun, Y., Wang, J.-W., You, C.-Q. & Sun, B. (2016). *J. Mater. Chem. C.* **4**, 8061–8069.

[bb9] Milek, M., Heinemann, F. W. & Khusniyarov, M. M. (2013). *Inorg. Chem.* **52**, 11585–11592. 10.1021/ic401960x 24063424

[bb10] Mörtel, M., Lindner, T., Scheurer, A., Heinemann, F. W. & Khusniyarov, M. M. (2020). *Inorg. Chem.* **59**, 2659–2666. 10.1021/acs.inorgchem.9b01424 31509384

[bb11] Mörtel, M., Witt, A., Heinemann, F. W., Bochmann, S., Bachmann, J. & Khusniyarov, M. M. (2017). *Inorg. Chem.* **56**, 13174–13186. 10.1021/acs.inorgchem.7b01952 29028322

[bb12] Naggert, H., Rudnik, J., Kipgen, L., Bernien, M., Nickel, F., Arruda, L. M., Kuch, W., Näther, C. & Tuczek, F. (2015). *J. Mater. Chem. C.* **3**, 7870–7877.

[bb13] Nihei, M., Suzuki, Y., Kimura, N., Kera, Y. & Oshio, H. (2013). *Chem. Eur. J.* **19**, 6946–6949. 10.1002/chem.201300767 23609989

[bb14] Ossinger, S., Kipgen, L., Naggert, H., Bernien, M., Britton, A. J., Nickel, F., Arruda, L. M., Kumberg, I., Engesser, T. A., Golias, E., Näther, C., Tuczek, F. & Kuch, W. (2020*a*). *J. Phys. Condens. Matter*, **32**, 114003. 10.1088/1361-648X/ab5c92 31778990

[bb15] Ossinger, S., Naggert, H., Bill, E., Näther, C. & Tuczek, F. (2019). *Inorg. Chem.* **58**, 12873–12887. 10.1021/acs.inorgchem.9b01813 31525895

[bb16] Ossinger, S., Näther, C. & Tuczek, F. (2016). *IUCrData*, **1**, x161252.

[bb17] Ossinger, S., Näther, C. & Tuczek, F. (2020*b*). *J. Phys. Condens. Matter*, **32**, 094001. 10.1088/1361-648X/ab5776 31722328

[bb18] Real, J. A., Muñoz, M. C., Faus, J. & Solans, X. (1997). *Inorg. Chem.* **36**, 3008–3013. 10.1021/ic960965c 11669951

[bb19] Ru, J., Yu, F., Shi, P.-P., Jiao, C.-Q., Li, C.-H., Xiong, R.-G., Liu, T., Kurmoo, M. & Zuo, J.-L. (2017). *Eur. J. Inorg. Chem.* **2017**, 3144–3149.

[bb20] Ruben, M. & Kumar, K. S. (2019). *Angew. Chem. Int. Ed.* **10**, https://doi.org/10.1002/anie.201911256.

[bb21] Sheldrick, G. M. (2015*a*). *Acta Cryst.* A**71**, 3–8.

[bb22] Sheldrick, G. M. (2015*b*). *Acta Cryst.* C**71**, 3–8.

[bb23] Spek, A. L. (2015). *Acta Cryst.* C**71**, 9–18. 10.1107/S2053229614024929 25567569

[bb24] Spek, A. L. (2020). *Acta Cryst.* E**76**, 1–11. 10.1107/S2056989019016244 PMC6944088 31921444

[bb25] Stoe & Cie (2008). *X-AREA*. Stoe & Cie, Darmstadt, Germany.

[bb26] Thompson, A. L., Goeta, A. E., Real, J. A., Galet, A. & Carmen Muñoz, M. (2004). *Chem. Commun.* pp. 1390–1391. 10.1039/b403179b 15179479

[bb27] Westrip, S. P. (2010). *J. Appl. Cryst.* **43**, 920–925.

[bb28] Xue, S., Guo, Y., Rotaru, A., Müller-Bunz, H., Morgan, G. G., Trzop, E., Collet, E., Oláh, J. & Garcia, Y. (2018). *Inorg. Chem.* **57**, 9880–9891. 10.1021/acs.inorgchem.8b00850 30080034

