# Supplementary figures and images for: Crystal structure of bis(5-bromo-1,10-phenanthroline-κ2 N,N′)bis­[di­hydro­bis­(pyrazol-1-yl)borato-κ2 N 2,N 2′]iron(II) toluene disolvate
Source: Acta Crystallogr E Crystallogr Commun. 2020 Jul 31;76(Pt 8):1398–402. doi: 10.1107/S2056989020010361 (PMC7405586; doi:10.1107/S2056989020010361)

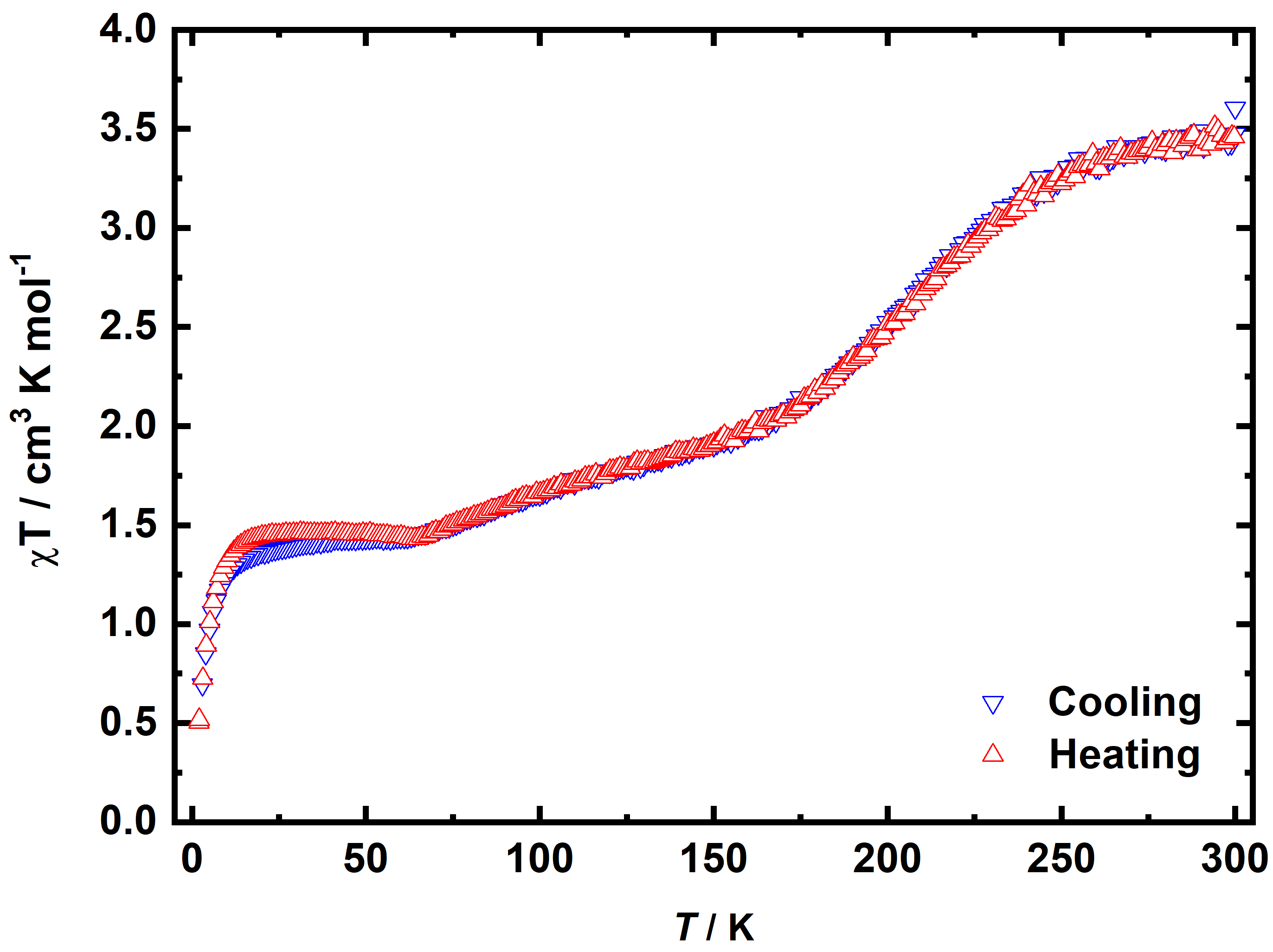

Supplement: Supplementary file 3 [file e-76-01398-sup3.png]

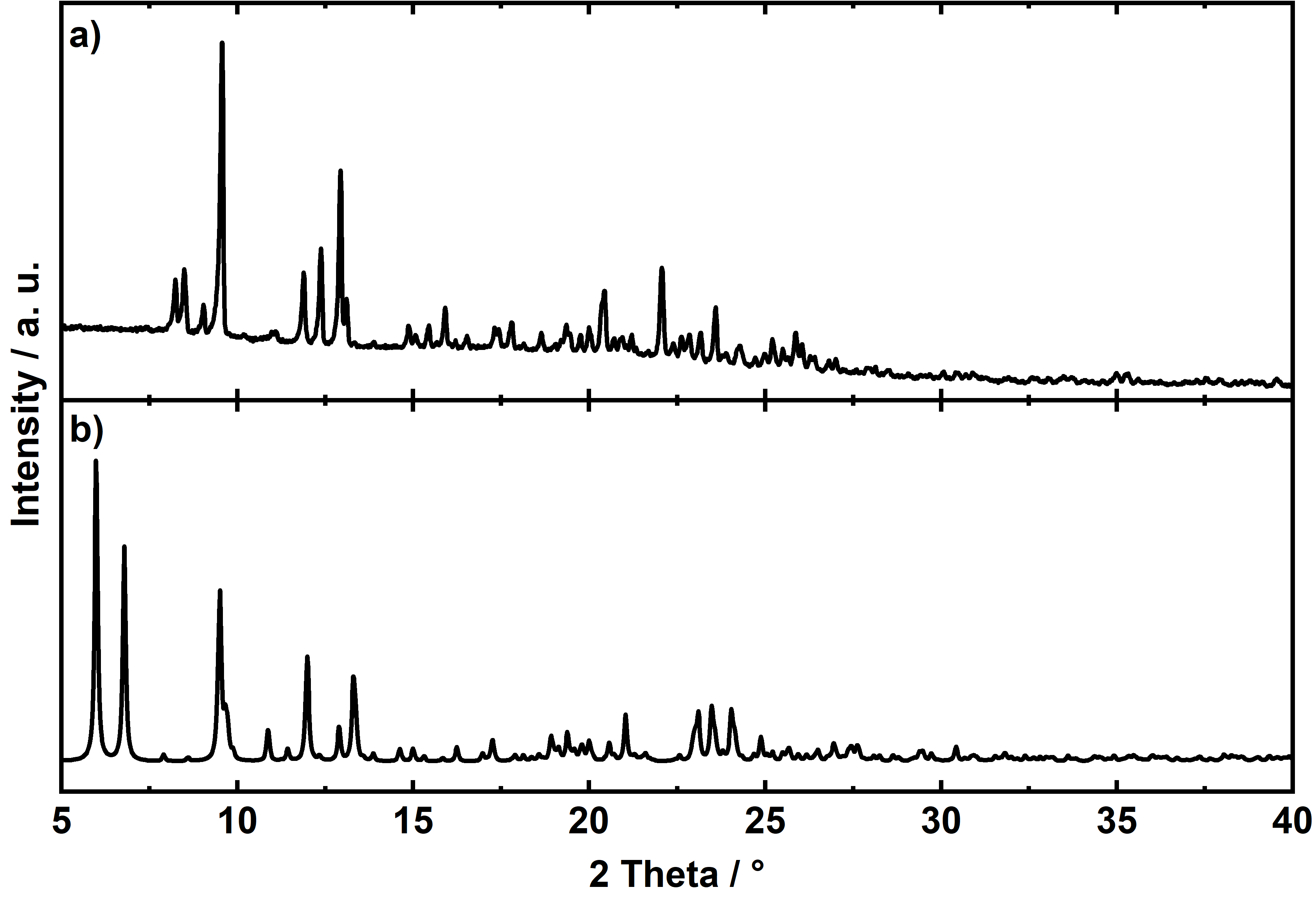

Supplement: Supplementary file 4 [file e-76-01398-sup4.png]
